# Changes in hair cortisol during retirement transition: the Finnish retirement and aging study

**DOI:** 10.1016/j.cpnec.2025.100325

**Published:** 2025-11-04

**Authors:** Konsta Kuusento, Susanna Kortesluoma, Saana Myllyntausta, Jussi Vahtera, Linnea Karlsson, Sari Stenholm

**Affiliations:** a Department of Public Health, University of Turku and Turku University Hospital, Turku, Finland; b Centre for Population Health Research, University of Turku and Turku University Hospital, Turku, Finland; c FinnBrain Birth Cohort Study, Turku Brain and Mind Center, Department of Clinical Medicine, University of Turku and Turku University Hospital, Turku, Finland; d Department of Psychology and Speech-Language Pathology, University of Turku, Turku, Finland; e Department of Child Psychiatry, Turku University Hospital and University of Turku, Turku, Finland; f Research Services, Turku University Hospital and University of Turku, Turku, Finland

**Keywords:** Hair cortisol, Stress, Work, Retirement, Cohort study

## Abstract

**Objectives:**

Retirement is a significant life event involving removal of work stress and changes in other psychological factors. These changes may induce physiological responses in the body, such as changes in levels of the stress hormone cortisol, but no previous studies exist on the topic. The aim of this study was to examine changes in hair cortisol concentration and the associated work-related factors during the retirement transition.

**Methods:**

One hundred and ninety-nine workers from the Finnish Retirement and Aging study participated in annual hair sampling before and after the retirement transition. Hair cortisol concentration was measured using mass spectrometry. Work-related factors were examined through survey measures. Latent trajectory analysis was used to investigate the heterogeneity of the changes in hair cortisol concentration during the retirement transition.

**Results:**

The study population had a mean age of 63.1 (SD 1.1) years and 92 % were women. Three trajectory groups for hair cortisol changes were identified: ”stable low” (79 %), “fluctuating” (6 %) and “post-retirement increase” (15 %). ”Post-retirement increase” group consisted of older participants compared to the “stable low” and “fluctuating” groups. No significant differences in exposure to work-related stressors were found between the groups.

**Conclusion:**

For the majority of the participants, the levels of hair cortisol remained relatively low and stable during the retirement transition, but for subgroups of individuals annual fluctuation and post-retirement increase in levels of hair cortisol were observed. Work-related stressors were not found to explain the variability, thus further research on changes in hair cortisol changes during retirement is warranted.

## Highlights

• Hair cortisol changes have not been studied before during retirement transition.
• For most, hair cortisol levels remain low through the retirement transition.
• Marked changes in hair cortisol level are observed in a subgroup of retirees.
• Work-related stressors do not associate with hair cortisol level changes.

## 1 Introduction

Moving into retirement is a major transitional phase in late middle age concerning most people. Retirement is often accompanied by changes in various aspects of life due to changes in time use, social connections, and daily activities [1]. Many people find retirement to be a positive life event to look forward to. Retirement brings freedom and increases spare time in one's life when the hours tied to work and work-related stress are removed. People have more time to focus on family, hobbies, and other enjoyable activities after retirement. On the other hand, prior to retirement, employment usually provides structure, meaning, higher level of stable income, social contacts, and fulfillment in a person's life. Hence, retirement can also be a stressful event financially, socially, and emotionally as for example income and social relationships may change [2].

Work-related stressors naturally decrease after retirement, but social and other cumulative stressors may have longer and more lasting effects on mental health. Previous studies have shown that retirement may have many beneficial effects, especially on perceived health. These include a decrease in psychological stress [3], mental and physical fatigue, depression symptoms [4,5], and sleep difficulties [6] as well as improved self-rated health [7], quality of life and a feeling of autonomy [4]. Moreover, changes in more objective health indicators have also been reported, including a decrease in antidepressant use [8] and beneficial changes in awake ambulatory blood pressure [9], though no change in the risk of major chronic diseases has been observed [5]. However, the effects of retirement may vary depending on work characteristics and life circumstances, which are therefore important to consider [10]. Indeed, work stress as indicated by, for example high physical and psychological job demands and low job satisfaction during the later years of work have been shown to be associated with a steep increase in suboptimal health and poor sleep before retirement and greater benefits from retirement [7,11]. Until now, changes in stress and stress responses have mainly been assessed with questionnaires and interviews portraying a self-reported viewpoint on pre-retirement stress factors and how people react to retirement, and therefore there is a lack of research examining changes with physiological measures.

The activity of the Hypothalamic-Pituitary-Adrenal (HPA) axis has been a key subject in stress research. The axis’ end product, cortisol, is a glucocorticoid hormone synthesized in the adrenal cortex. Cortisol secretion is regulated downstream by the hypothalamic secreted corticotropin-releasing hormone (CRH) and the pituitary secreted adrenocorticotropic hormone (ACTH). Cortisol is released in response to physical and/or emotional stress thus it has also been called “the stress hormone” [12]. Cortisol can be traditionally measured from urine, serum, and saliva. The measured levels fluctuate during the day following the circadian rhythm and reacting to stress [13]. The HPA axis is a complex system and both hyper-an hypoactivity of the HPA axis has been reported in long-term stress and adversity [14,15]. Measurement of hair cortisol concentrations (HCC) is a relatively recent method to assess longer-term cumulative activity of the HPA axis. Elevated HCC has been associated with chronic stress especially if the stress is ongoing during the sampling. Meanwhile decreased HCC has also been found in people with generalized anxiety disorder and post-traumatic stress disorder. However, HCC is not viewed as a simple, direct way to quantify stress or stress exposure. It only captures one aspect of the endocrine activity of the HPA axis [16]. Despite increasing use of HCC as a stress-related biomarker in population-based studies, there is a scarcity of studies examining HCC and its changes in relation to work status or work stressors in a longitudinal setting. Most of the longitudinal studies with repeated HCC measurements have been conducted among maternal and children samples [[17], [18], [19], [20], [21], [22], [23], [24]].

To the best of our knowledge, only three previous studies have examined work-related stressors and changes in HCC in a longitudinal setting [18,25,26]. Among a sample of 229 Chinese fishermen (mean age 35.2 [SD 11.0]), significantly lower levels of HCC were observed over the past month at sea compared to the period of preparation [25]. It should be noted that this study used only one hair sample, divided into segments representing the time spent preparing for a fishing season on the sea compared to the time over the past one month at sea. The second study assessed HCC based on hair samples taken every fourth month among relatively young people starting their medical internships (n = 74) (mean age 27.4 [SD 2.4]). HCC was observed to increase sharply at the beginning of the internship, then decrease as the internship continued, and increase again at the end of the internship year. HCC did not correlate with depressive symptoms, hours worked, hours slept, or self-reported stress [18]. The third study by Herr et al. [26], which included forty male factory workers (mean age 47.9 [SD 6.3]), found an increase in work stress, measured by Effort-Reward-Imbalance, model to be associated with an increase in HCC during a one-year follow-up.

Work-related stressors are a part of employment for many employees. They differ in incidence and intensity between individuals, and they can trigger various physiological and psychological responses [27]. As work-related stressors are naturally withdrawn after transitioning into retirement, it could be hypothesized that work-related stressors prior to retirement could be associated with pre- and post-retirement levels of HCC. To the best of our knowledge, there are no previous studies examining longitudinal changes in HCC in older adults during the retirement transition.

To provide further insight into HPA axis activity and stress during the transition to retirement, the aim of this study was, for the first time, to examine changes in HCC during the retirement transition by utilizing annual repeated measurements of HCC before and after the retirement transition. In addition, the work-related factors associated with the observed HCC changes were examined. This study was designed to increase our understanding of the changes in physiological biomarkers, and underlying factors during the shift from employment to retirement.

## 2 Methods

### 2.1 Study design and participants

The Finnish Retirement and Aging Study (FIREA) (n = 6783) is an ongoing longitudinal cohort study established in 2013 consisting of public sector workers who have been followed up from the final years of employment through the retirement transition and to post-retirement years. Details of the design and implementation of the FIREA study have been reported elsewhere [28]. Shortly, FIREA cohort participants received the first questionnaire 18 months prior to the estimated retirement age. After responding to the questionnaire, Finnish-speaking participants with an estimated retirement date between 2017 and 2019, who lived in Southwest Finland and were still working, were invited to participate in a clinical sub-study (n = 773). Of them, 290 agreed to participate. Thereafter, the study participants were followed up with annual measurements including, for example, hair and blood samples, cognitive function tests, cardiovascular function tests, and questionnaires. To determine the timing of retirement, the actual retirement day was quired during each phase of the data collection, and this information was used to determine pre- and post-retirement measurements. The annual data points were named to represent the time before retirement (−2 and −1) and time after retirement (+1 and + 2) with the actual retirement date falling between the data points of −1 and +1. Every measure was conducted by the same practice at each data point.

For the current study, we included clinical sub-study participants (n = 199), who had provided at least one hair sample before and at least one after the transition to full-time statutory retirement. The number of HCC measures ranged from 2 to 4 per participant. The majority of participants provided three samples (n = 157), 14 participants provided the maximum amount of four samples, 28 participants provided two samples.

FIREA study was conducted according to the Declaration of Helsinki and approved by the Ethics Committee of the Hospital District of Southwest Finland.

### 2.2 Assessment of hair cortisol concentration

Hair samples and hair treatment related information were collected during the clinical visit by a study nurse. The visits were conducted always at the same time of the year to reduce the possibility of seasonal changes in HCC due to UV light exposure [29]. A 3-cm segment of proximal hair was cut from a standardized area of the posterior vertex as close as the scalp as possible. After collecting the hair was protected from light and moisture with foil. Hair cortisol concentrations were analyzed using method of LC-MS/MS mass spectrometry in Technical University of Dresden, Germany [30]. Each sampling and analysis were done using the same method.

The participants also filled in a survey regarding hair treatments, hair characteristics, and hormonal medications each time when the sampling was conducted. Hair treatment such as the hair washing frequency has previously been associated with hair cortisol [16]. After examination, there was no association with hair treatments, hair characteristics, or hormonal medications with the trajectory groups. Hair washing frequency variable was dichotomized (0–3 times per week and 4+ times per week).

### 2.3 Assessment of work-related factors

Occupational status was obtained from the register of the pension institute Keva. Occupations were coded in line with the International Standard Classification of Occupations (ISCO) and categorized into two groups: high – intermediate (ISCO classification 1–4, managers, professionals, office workers) and low (ISCO classification 5–9, manual and service workers) [[31], [34]].

Information on work-related stressors (job strain, shift work, and work time control) was collected with a survey questionnaire. For the analyses, we used information from the last survey available before retirement.

Job strain was assessed with the Job Content Questionnaire [32]. Nine items of job control and five items of job demands were scaled on a five-step scale to represent individual levels of job demands and job control. The participants were categorized as high vs. low demands and high vs. low control based on median scores in the sample (3.40 for demands and 3.76 for control). Using the demand-control model, participants who had both high demands and low control were included in the high strain group, while the other participants were included in the low strain group [33,34].

Work time control was assessed by asking participants to rate, on a scale from 1 (very little) to 5 (very much), how much they could influence the following aspects of their workday: length of a workday, the starting and ending times of a workday, the taking of breaks during the workday, the handling of private matters during the workday, the scheduling of work shifts, the scheduling of vacations and paid days off, and the taking of unpaid leave [35]. Participants whose combined scores placed within the lowest quartile were classified as having low work time control, while those in the remaining three quartiles were categorized as high work time control. This divide has been used in previous FIREA studies [36].

Shift work was determined by asking the participants to describe their typical work time mode. Participants were categorized into no shift work (regular daytime work) and shift work (shift work without night shifts, shiftwork with night shifts, regular nighttime work, and other irregular work).

Work related stressors tend to cluster on the same individuals [37]. Hence, we formed a summary variable indicating the number of work-related stressors (high job strain, low work time control, shift work) the participants were exposed to. The participants were categorized into having any work-related stressor or none.

### 2.4 Assessment of other factors

Sex and date of birth were obtained from the register of the pension institute Keva. Other characteristics were obtained from the last questionnaires preceding the transition to retirement. Marital status was dichotomized as married/cohabitating and never married/divorced/separated/widowed. Self-reported health was dichotomized into good (good/rather good) and suboptimal (average/rather poor/poor) as in previous studies [38]. The Body-Mass Index was derived from measured weight and height during the study visits.

Alcohol consumption and smoking status were obtained from the survey data. Alcohol risk usage (no vs yes; >24 units for men and >16 units for women) [39] and smoking status (no vs yes) were dichotomized. Alcohol risk usage has been shown to associate with HPA axis dysregulation [40] and smoking with the activity of HPA axis [41]. After examinations in our data, only a small number of participants reported current smoking (5 %) or alcohol risk use (3 %), and they were not associated with HCC trajectories. Therefore, alcohol use and smoking were not included in the main analysis.

### 2.5 Statistical analysis

Because of the skewed distribution of HCC, natural logarithm (ln) conversion was performed, and they were used in the analysis. Because extreme values of HCC are quite common, we conducted careful examination of potential outliers by each study wave separately. There were 8 sporadic values exceeding three standard deviations (SD) above the mean and these values were excluded from the analysis, but other observations from the participants were included in the analysis.

Descriptive information on participant characteristics of the total study population and by the trajectory groups are presented using means and standard deviations for continuous variables and frequencies and percentages for categorical variables.

To illustrate changes in HCC at the population level during the retirement transition, we used linear regression analyses with generalized estimating equations (GEEs). The results are shown as mean values and 95 % confidence intervals (CI).

To investigate the heterogeneity of the changes in HCC during the retirement transition, latent trajectory analysis (PROC TRAJ in SAS 9.4) was used. We used Nagin's 2-step procedure to determine the optimal number trajectories and chose the number and order of regression parameters [42]. In the first step, we fitted an increasing number of trajectory models with curvilinear polynomial shape for HCC until no improvement in model fit was observed. Assessment of model fit was based on Bayesian information criterion (BIC) values, Akaike information criterion (AIC) values, Log-likelihood, and posterior probabilities. In the second step, we tested models with quadratic and linear trajectories for the selected models chosen in the first step. The best-fitting trajectory model was chosen and used as a grouping variable in the following analytical stage. The model fit statistics of the latent trajectory analyses from polynomial models with 1–4 trajectories for hair cortisol concentrations can be found in the Supplementary Table 1.

A multinomial logistic regression analysis was used to examine the associations between categorical sociodemographic, work- and health-related factors and probability of being classified into a particular trajectory group. The analyses were first adjusted for age and sex (Model 1), and thereafter additionally for self-reported health, hair washing frequency and occupational status (Model 2).

The analyses were conducted using SAS 9.4 software [SAS institute, Cary NC].

## 3 Results

The characteristics of study participants before retirement (study wave −1) are presented in Table 1. Mean age of participants was 62.2 (SD 1.1) years and majority were women (92 %). Regarding work-related factors, one third (32 %) worked in manual or service occupations, and about one fifth reported high job strain (22 %) and low work time control (22 %). In addition, 28 % did shift work. Half of the study population had at least one work-related stressor (50 %) and 17 % had a clustering of two or three stressors.

**Table 1:** Descriptive characteristics of the participants by the hair cortisol concentration trajectory groups.

	Total study population n = 199	Stable low n = 159	Fluctuating n = 11	Post-retirement increase n = 29	
	Mean (SD)	Mean (SD)	Mean (SD)	Mean (SD)	p-value

Age, years	63.2 (1.1)	63.1 (1.1)	63.0 (0.8)	63.7 (1.0)	0.03
Body Mass Index, kg/m^2^	26.3 (4.8)	26.2 (5.0)	26.9 (3.9)	26.5 (4.3)	0.87

	n (%)	n (%)	n (%)	n (%)	p-value

Sex					0.60
Male	15 (9)	13 (8)	0 (0)	2 (7)	
Female	184 (92)	146 (92)	11 (100)	27 (93)	
Occupational status					0.60
High and intermediate	136 (68)	107 (67)	9 (82)	20 (69)	
Low	63 (32)	52 (33)	2 (18)	9 (31)	
Marital status					0.04
Married or cohabitant	132 (66)	99 (62)	8 (73)	25 (86)	
Single, divorced or widow	58 (29)	52 (33)	3 (27)	3 (10)	
Missing	9 (5)	8 (5)	0 (0)	1 (3)	
Self reported health					0.22
Good	152 (76)	125 (79)	8 (73)	19 (66)	
Suboptimal	40 (20)	28 (18)	3 (27)	9 (31)	
Missing	7 (4)	6 (4)	0 (0)	1 (3)	
Smoking					0.36
No	181 (91)	115 (72)	6 (55)	17 (59)	
Yes	9 (5)	37 (23)	5 (45)	12 (41)	
Missing	9 (5)	9 (5)	0 (0)	0 (0)	
Alcohol riskuse					0.27
No	188 (94)	149 (94)	10 (91)	29 (100)	
Yes	5 (3)	4 (3)	1 (9)	0 (0)	
Missing	6 (3)	6 (3)	0 (0)	0 (0)	
Job strain					0.51
Low strain	151 (76)	118 (74)	10 (91)	23 (79)	
High strain	44 (22)	37 (23)	1 (9)	6 (21)	
Missing	4 (2)	4 (3)	0 (0)	0 (0)	
Work Time control					0.82
High control	144 (72)	113 (71)	9 (82)	22 (76)	
Low control	41 (21)	34 (21)	2 (18)	5 (17)	
Missing	14 (7)	12 (8)	0 (0)	2 (7)	
Shift work					0.34
No	140 (70)	109 (69)	10 (91)	21 (72)	
Yes	55 (28)	46 (29)	1 (9)	8 (28)	
Missing	4 (2)	4 (3)	0 (0)	0 (0)	
Any Work-Related Stressor					0.28
No	96 (48)	74 (47)	8 (72)	14 (48)	
Yes	99 (50)	81 (51)	3 (27)	15 (52)	
Missing	4 (2)	4 (3)	0 (0)	0 (0)	

Notes: SD = Standard Deviation.

The characteristics of the final study sample compared to the entire clinical substudy sample, and the survey study sample are presented in Supplementary Table 2. Compared to the entire clinical substudy sample, the final study sample consisted of more women. This was due to the difficulties of hair sampling due to very short hair in some male participants. Compared to the survey study sample, the final study sample also presented slightly better self-reported health, and better Work-Time Control. Regarding age, occupational status, marital status, alcohol use and other work-related stressors the different samples were very similar.

**Fig. 1:**
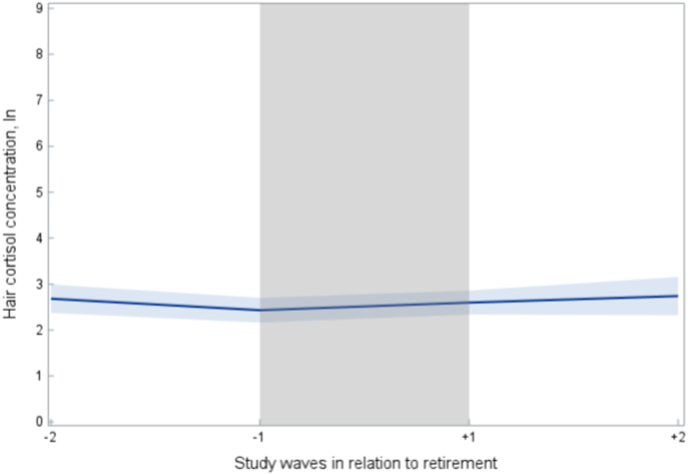
Mean Levels of Hair Cortisol Concentration During Retirement Transition. Shaded blue area represents the 95 % Confidence Interval. The grey area represents the period during which the retirement date occurred.

First, we examined the population mean levels of HCC during the years before and after retirement transition (Fig. 1). The average HCC level stayed relatively low and constant during the entire follow-up period and no marked change was observed during the retirement transition years. Mean levels of hair cortisol in original units (picogram/milligram) are shown in Supplemental Fig. 2.

**Fig. 2:**
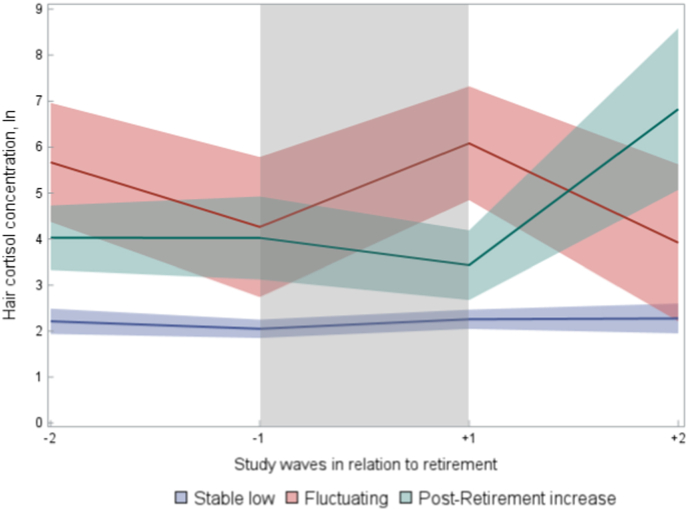
Trajectories of Hair Cortisol Concentration During Retirement Transition. Shaded colored area represents the 95 % Confidence Interval. The grey area represents the period during which the retirement date occurred.

Next, we examined heterogeneity in the HCC changes during the retirement transition by using latent trajectory analysis. Three different trajectories were identified, and they are shown in Fig. 2. The largest trajectory was “stable low” (79 %) showing low HCC before and after retirement. A slight increase in HCC occurred between study waves −1 and +1 (HCC 2.05 [95 %CI 1.85–2.25] vs. 2.26 [95 %CI 2.05–2.47]). The second-largest trajectory was “post-retirement increase” (15 %) showing a noticeable increase (HCC 3.43 [95 %CI 2.68–4.19) vs. 6.82 [95 %CI 5.07–8.58] in HCC during the post-retirement years (study waves +1 and + 2). The level of HCC was also higher compared to the “stable low” group. The third and smallest trajectory was “fluctuating” (6 %) in which the HCC level presented a decrease before retirement (study waves −2 to −1) (HCC 5.67 [95 %CI 4.37–6.96] vs. 4.26 [95 %CI 2.74–5.79]), increase during retirement transition (study waves −1 and +1) (4.26 [95 %CI 2.74–5.79] vs. 6.08 [95 %CI 4.84–7.31]) and again decrease post-retirement (study waves +1 to +2) (HCC 6.08 [95 %CI 4.84–7.31] vs. 3.92 [95 %CI 2.21–5.63]). The level was also higher than the “stable low” groups. Predicted probabilities of trajectory group membership ranged from 0.80 to 0.96.

Table 1 presents the characteristics of the three different trajectory groups. The “post-retirement increase” group had a slightly higher mean age, and marriage or cohabitation was more common than in the “stable low” group. There was no statistically significant difference in sex, occupational status group or health-related indicators between the trajectory groups. Job strain and shift work tended to be more common in the “stable low” and “post-retirement increase” groups than in the “fluctuating” group, but work-time control was similar across the groups.

Finally, we examined the associations between pre-retirement work-related factors and HCC trajectory groups (Table 2). There was a tendency towards lower odds for low occupational status, high job strain, low work time control, and shift work in “fluctuating” group compared to the reference group “stable low”. However, none of the factors reached statistical significance in either of the models. “Post-retirement increase” group showed no difference to “stable low” group in terms of the work-related factors.

**Table 2:** Association of work-related stressors with hair cortisol concentration trajectory groups.

	Stable low (Ref)	Fluctuating		Post-retirement increase	
	Odds Ratio	Odds Ratio	95 % CI	Odds Ratio	95 % CI
Occupational status (Low vs. High/Intermediate)					
Model 1	1	0.41	0.08–1.99	1.05	0.43–2.54
Model 2	1	0.46	0.09–2.34	0.95	0.36–2.43
Job strain (High vs Low)					
Model 1	1	0.32	0.04–2.58	0.70	0.24–2.01
Model 2	1	0.34	0.04–2.94	0.67	0.22–2.02
Work Time Control (Low vs High)					
Model 1	1	0.66	0.14–3.23	0.86	0.29–2.53
Model 2	1	0.79	0.15–4.05	0.81	0.26–2.52
Shift work (Yes vs No)					
Model 1	1	0.22	0.03–1.78	0.96	0.39–2.41
Model 2	1	0.26	0.03–2.17	0.94	0.35–2.48
Any Work-related stressor (Yes vs No)					
Model 1	1	0.30	0.08–1.21	1.11	0.49–2.52
Model 2	1	aNot estimable	Not estimable	0.61	0.17–2.17

Notes: Model 1 adjusted for age and sex. Model 2 adjusted additionally for self-reported health, body mass index, hair washing frequency and occupational status. CI = Confidence Interval.

a The regression model did not provide a reliable estimate due to small sample size (infinite confidence intervals).

## 4 Discussion

In this longitudinal study, we examined changes in HCC among Finnish public sector employees during the surrounding years of retirement. Overall, no marked change was observed in HCC during the follow-up, when examining the average values in the study population. However, when applying latent trajectory analysis, we identified three different trajectory groups for HCC. Most of the participants (79 %) were assigned to the group presenting stable and low levels of HCC both before and after retirement. The second largest group (15 %) showed higher levels of HCC before and after retirement, and was characterized by a sharp increase in HCC after retirement. The smallest group (6 %) presented fluctuating levels of HCC, with decreasing levels before and after retirement, but increasing levels during the retirement transition. We found no associations between work-related stressors measured before retirement and the trajectory groups.

To our knowledge, this is the first study examining HCC longitudinally during the surrounding years of retirement. Although the majority of the participants showed stable and low levels of HCC during the transition, an interesting finding was that about a fifth of the participants showed either fluctuation or post-retirement increase in HCC. This corroborates earlier findings about heterogeneity in individuals’ responses and adaptation to retirement [3,38].

As it has been previously shown that retirement decreases the risk of depression [43], decreases psychological stress [3] and increases the feeling of autonomy [4], we hypothesized that this would also be observed in physiological biomarkers such as HCC. We also hypothesized that the removal of work-related stressors, particularly the clustering of work-related stressors, would result in a decrease in HCC following retirement. However, we found no trajectory with a constant decrease in HCC following retirement. In addition, work-related stressors tended to be less common in the trajectory groups presenting higher levels and more fluctuation of HCC. Despite the tendency, there were no significant differences between the groups regarding work-related stressors, which may be partly due to the small sample size and should be further studied in larger study populations.

However, our findings are in line with a previous cross-sectional study on older adults (mean age 68.1 [SD 5.3]) from England, which also found no association with HCC and employment status and perceived stress [44]. On the other hand, in another cross-sectional study from the United States among participants with cardiovascular disease from various occupations (mean age 55.5 [SD 13.5]) an association between work-related factors, namely regarding more support from co-workers or supervisors and better work-life balance, and lower HCC was found [45].

We are aware of only three previous longitudinal studies examining work-related factors and HCC [18,25,26]. Study by Herr et al. [26] conducted among middle-aged male factory workers observed concurrent changes in work-related stress and HCC. Studies by Wu et al. [25] and Mayer et al. [18] found an increase in HCC sharply after the stressor onset or even before the onset possibly due to the anticipation of the stressor. The anticipation of retirement might be portrayed as the pre-retirement increase of HCC in the “Fluctuating” trajectory group, but this phenomenon was not observed in other trajectory groups. These findings are somewhat in contrast to our findings, because we did not observe an association between work-related stressors and HCC trajectory groups. However, direct comparison of our results to previous studies is difficult due to differences in study populations, study designs, and occupations.

When interpreting the findings, it is also important to consider physiological age-related changes in cortisol concentration. A recent study from 20- to 90-year-old volunteers from the Baltimore Longitudinal Study of Aging reported that urinary free cortisol excretion exhibits a U-shaped pattern during adulthood [46]. Cortisol secretion somewhat increases after the age of 50, and the increase persists into the early 60s, which represents the age group of our study. When the overall pattern of cortisol secretion is increasing with age, this could explain why we could not find trajectories with decreasing HCC, even though the hypothetical stress-relieving transition to retirement occurred. Aging can also result in HPA axis dysregulation manifesting as a flatter diurnal cortisol pattern [47]. The dysregulation may cause attenuated physiological responses even though the life transition itself is meaningful and potentially stressful or stress relieving.

In addition to the age-related changes, the intrinsic negative feedback and central “top-down” regulation of the HPA axis continually act to stabilize cortisol secretion within restricted limits, preserving reactivity to novel stimuli while diminishing responses to recurrent or familiar stressors [12]. It is possible that retirement might be more familiar stressor than we anticipated, or the possible activity of the HPA axis was regulated rapidly, and our timing of the hair samples did not record it.

A major strength of this study was the repeated annual measurements of HCC in a unique setting before and after retirement. We collected each yearly hair sample individually and did not use different segments of one hair sample. We had a broad representation of occupations from each class of International Standard Classification of Occupations, which broadens the generalizability compared to studies focusing on one occupation. We also had a wide range of information regarding the characteristics of individuals’ work and work-related stressors. We were able to analyze whether the removal of these factors would be associated with the changes in HCC while controlling for various confounders.

The main limitation of this study was a relatively small sample size, which is however comparable to previous studies on HCC changes in different occupation groups. The identified trajectory groups were small, but still distinct from each other as suggested by the model fit statistics. The small sample may also limit the power to identify associations between work-related stressors and trajectory groups. The generalizability of our findings may be limited, as the participants were mainly women and relatively healthy public sector employees of European origin in a Nordic welfare state. The analysis comparing the final study sample to the substudy sample and the survey study sample strengthens the generalizability. High work stress was uncommon, as almost half (48 %) of the population was not exposed to any of the stressors examined. The work-related stressors were obtained from the last questionnaire completed before retirement and did not represent the possible accumulation of stress during the whole career. Further research is needed to examine whether similar findings are found in other labor market sectors, countries, and cultures. Larger sample sizes should be incorporated to reach more statistical power. Longer follow-up periods could help to understand how HCC changes in the long term during post-retirement. Standardized timing of the hair sampling in relation to the actual retirement date could better capture the possible changes in HCC. Examining other possible factors (e.g., coping methods, resilience, support from family and friends) associated with HPA axis activity could help explain the changes in HCC during the retirement period. Other physiological markers of stress e.g., C-reactive protein or catecholamines could be utilized [48]. In addition, the allostatic load model combining multiple physiological markers could be used [49].

In conclusion, HCC remains relatively stable for a large majority of participants undergoing retirement transition in Finland. In addition, for some participants, HCC shows a temporal increase during retirement or an increase during the post-retirement years. Work-related stressors did not explain differences in HCC changes, and thus, further research on the potential factors predicting HCC changes is warranted.

## CRediT authorship contribution statement

**Konsta Kuusento:** Writing – review & editing, Writing – original draft, Methodology, Funding acquisition, Formal analysis, Conceptualization. **Susanna Kortesluoma:** Writing – original draft, Methodology, Conceptualization. **Saana Myllyntausta:** Writing – review & editing, Writing – original draft. **Jussi Vahtera:** Writing – review & editing, Writing – original draft. **Linnea Karlsson:** Writing – review & editing, Writing – original draft, Supervision, Methodology, Conceptualization. **Sari Stenholm:** Writing – review & editing, Writing – original draft, Supervision, Resources, Methodology, Formal analysis, Conceptualization.

## Declaration of competing interest

The authors declare the following financial interests/personal relationships which may be considered as potential competing interests: Konsta Kuusento reports financial support was provided by Tyks and 10.13039/501100009420Hospital District of Southwest Finland. Sari Stenholm reports financial support was provided by Research Council of Finland. Sari Stenholm reports financial support was provided by Finnish Ministry of Education and Culture. Sari Stenholm reports financial support was provided by 10.13039/501100004325Signe and Ane Gyllenberg Foundation. Sari Stenholm reports financial support was provided by Juho Vainio Fundation. Konsta Kuusento reports financial support was provided by 10.13039/501100005609University of Turku University Hospital Center for Education and Research. Susanna Kortesluoma reports financial support was provided by Yrjö Jahnsson Foundation. If there are other authors, they declare that they have no known competing financial interests or personal relationships that could have appeared to influence the work reported in this paper.
