# Supplementary material for: Changes in hair cortisol during retirement transition: the Finnish retirement and aging study
Source: Compr Psychoneuroendocrinol. 2025 Nov 4;24:100325. doi: 10.1016/j.cpnec.2025.100325 (PMC12861725; doi:10.1016/j.cpnec.2025.100325)

**Supplementary material.**

**Supplemental Table 1.** Model fit statistics of the latent trajectory analyses from polynomial models with 1 to 4 trajectories for hair cortisol concentrations

| Number of trajectories | Polynomical order | BIC | AIC | Log-likelihood | Average posterior probabilities | Smallest group (%) |
| --- | --- | --- | --- | --- | --- | --- |
| 1 | 3 | -1041.52 | -1033.29 | -1028.29 | 1 | 100 |
| 2 | 33 | -1000.38 | -983.91 | -973.91 | 0.970/0.877 | 17.9 |
| **3** | **333** | **-1000.01** | **-975.31** | **-960.31** | **0.956/0.895/0.795** | **6.4** |
| 4 | 3333 | -1002.24 | -969.3 | -949.3 | 0.949/0.964/0.998/0.879 | 2.0 |

Notes: BIC = Bayesian information criterion values, AIC = Akaike information criterion values.

**Supplemental Table 2.** Pre-retirement characteristics of the study sample included in the analyses (n=199), clinical substudy sample (n=269) and survey study sample (n=3948) in the last available measurement in which the participants were still working.

|  | Current study sample  (n=199) |  | Clinical substudy sample  (n=269) | Survey study sample  (n=3948) |
| --- | --- | --- | --- | --- |
|  | Mean (SD) |  | Mean (SD) | Mean (SD) |
| Age, years | 63.2 (1.1) |  | 63.2 (1.1) | 63.4 (1.4) |
| Body Mass Index, kg/m^2^ | 26.3 (4.8) |  | 26.3 (4.7) | *26.8 (4.5) |
|  | n (%) |  | n (%) | n (%) |
| Sex, Female |  |  |  |  |
| Male | 15 (9) |  | 45 (17) | 684 (17) |
| Female | 184 (92) |  | 224 (83) | 3264 (82) |
| Occupational status |  |  |  |  |
| High and intermediate | 136 (68) |  | 185 (69) | 2521 (64) |
| Low | 63 (32) |  | 84 (31) | 1393 (35) |
| Missing | 0 (0) |  | 0 (0) | 34 (1) |
| Marital status |  |  |  |  |
| Married or cohabitant | 132 (66) |  | 178 (66) | 2514 (64) |
| Single, divorced or widow | 58 (29) |  | 76 (28) | 1018 (26) |
| Missing | 9 (5) |  | 15 (6) | 416 (11) |
| Self reported health |  |  |  |  |
| Good | 152 (76) |  | 206 (77) | 2744 (70) |
| Suboptimal | 40 (20) |  | 52 (19) | 877 (22) |
| Missing | 7 (4) |  | 11 (4) | 327 |
| Smoking |  |  |  |  |
| No | 181 (91) |  | 243 (90) | 3245 (82) |
| Yes | 9 (5) |  | 12 (4) | 311 (8) |
| Missing | 9 (5) |  | 14 (5) | 392 (8) |
| Alcohol riskuse |  |  |  |  |
| No | 188 (94) |  | 250 (93) | 3519 (89) |
| Yes | 5 (3) |  | 9 (3) | 91 (2) |
| Missing | 9 (5) |  | 10 (4) | 338 (9) |
| Job strain |  |  |  |  |
| Low strain | 151 (76) |  | 205 (76) | 2908 (74) |
| High strain | 44 (22) |  | 56 (21) | 718 (18) |
| Missing | 4 (2) |  | 8 (3) | 322 (8) |
| Work Time control |  |  |  |  |
| High control | 144 (72) |  | 194 (72) | 2234 (57) |
| Low control | 41 (21) |  | 54 (20) | 1091 (28) |
| Missing | 14 (7) |  | 21 (8) | 623 (16) |
| Shift work |  |  |  |  |
| No | 140 (70) |  | 198 (74) | 2747 (70) |
| Yes | 55 (28) |  | 64 (24) | 878 (22) |
| Missing | 4 (2) |  | 7 (3) | 323 (8) |

Notes: * Based on self-reported body weight and height.

**Supplemental Figure 1.** Flow chart for the selection of the study population.

Clinical sub-study participants n=290

**Finnish Retirement and Aging study (FIREA)** n=6783

Excluded:

Did not provide hair sample before and after retirement n=58

Excluded:

Hair cortisol concentration outliers n=8

Final analytical sample n=199

Provided hair sample both before and after retirement n=207

Participated at least two study waves, one before and one after retirement
n=265

Excluded:

Did not participate in clinical study visits before and after retirement n=25

**Supplemental Figure 2.** Mean Levels of Hair Cortisol Concentration During Retirement Transition in Raw Units. Shaded blue area represents the 95% Confidence Interval. The grey area represents the period during which the retirement date occurred.


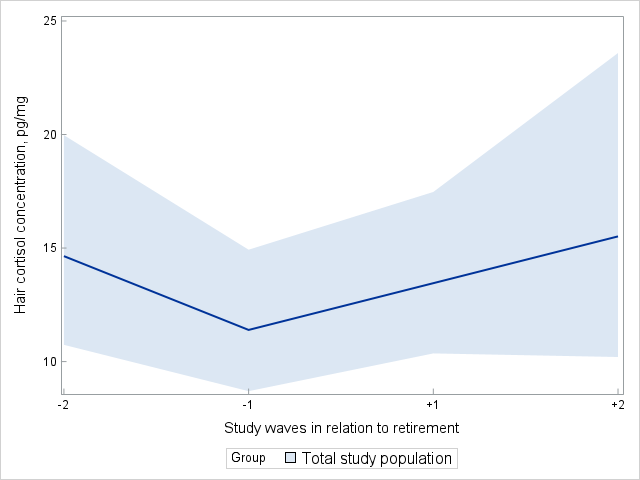

Supplement: Multimedia component 1 [file mmc1.docx]
